# Beyond Meat: Alternative Sources of Proteins to Feed the World

**DOI:** 10.3390/nu15132899

**Published:** 2023-06-27

**Authors:** Francesco La Barbera, Mario Amato, Fabio Verneau

**Affiliations:** Department of Political Science, University of Naples Federico II, Via Rodinò 22/a, 80138 Naples, Italy; mario.amato@unina.it (M.A.); verneau@unina.it (F.V.)

A number of changes and social factors such as the expected population growth, the raising demand for animal proteins, food chain disruption due to the COVID-19 pandemic and conflicts are placing food security and sustainable diet at the very centre of the political agenda. Food security is part of the “Zero Hunger” target of the 2030 Agenda for Sustainable Development Goals (SDGs) adopted by the United Nations (UN) in 2015 which aims “to end hunger, achieve food security, improve nutrition and promote sustainable agriculture” [1]. Considering that world population will reach almost 10 billion by 2050, the issue of securely feeding the world has never been more vital. Nevertheless, the mission to provide sufficient, safe, and nutritious food [1] has been repeatedly jeopardized over the last 15 years, undermining the myth of price stability and food availability itself. More recently, the 2020 COVID-19 pandemic [2] and the current spread of international conflicts have further threatened the world’s food security. Since February 2022, at the beginning of the conflict between the Russian Federation and Ukraine, wheat prices have risen by 30 percent; furthermore, the disruption of some strategic global food chains, such as wheat and sunflower, is expected to clash especially in Africa, which is already facing severe food insecurity problems [3,4]. In a similar fashion, the prices of conventional protein sources are constantly rising, further mining the two food security pillars of availability and access. Figure 1 shows the yearly average price of the three main conventional protein sources destined for human consumption—beef, poultry, and fish—and the main protein source destined for animal feeding—soybean meal. A number of important price spikes are clearly visible, underlining a mostly common pattern with sensitivity to the financial crisis of 2007/2008, the consequences of Arab Spring in 2010, the impact of the COVID-19 pandemic in 2021, and the Russian–Ukrainian conflict in 2022.

The second aspect highlighted in the literature is the environmental issue inherent to conventional animal protein production. There is a growing academic interest regarding the fact that meat production and consumption play an important role in environmental impact and climate change [5,6], where food systems are responsible for 1/3 of GHG emissions [7] and the production of food is required to double in the next years in order to satisfy the growing demand. Intensive livestock farming takes a major role in what has been described so far due to the land area used for feed crops (one third of all arable land) and the ammonia emissions from livestock manure [8,9]. Negative consequences also include biodiversity loss, disruption of ecosystems, air pollution, depletion of freshwater resources and soil quality [10]. This notwithstanding, developing countries and booming economies are increasing their consumption of meat and dairy products [9], whereas in Western Europe, there is a growing interest in alternative proteins [11]. Overall, according to FAO, the consumption of conventional animal origin proteins continues to grow, with particularly sharp dynamics in China and especially in India (see Figure 2).

The Special Issue “Beyond meat: Alternative sources of protein to feed the world” addresses several topics which can contribute to the ongoing research on possible alternatives to meat-based proteins.

The first point regards consumers’ acceptance of substitutes to traditional animal proteins. Paramount research in recent years has been devoted to this topic, showing that consumers are mostly skeptical about some possible alternative proteins such as those derived from insects. This is confirmed by the study conducted by Kamenidou and colleagues [12] with a large sample of Greek participants. Their results show that the young subjects who responded to their survey—all belonging to the so-called Generation Z—are generally not willing to substitute traditional meat proteins with insect-based alternatives. Nevertheless, they are quite familiar with the topic of entomophagy and show some signal of interest toward it, especially in relation to foods containing processed (non-visible) insects. Consumers’ skepticism is also observed by Amato and colleagues [13] in relation to innovative practices related to animal feeding. In their study, these scholars show an innovative application of the best-worst method.

As already mentioned, consumer attitudes and behaviors towards alternative proteins have been extensively studied in recent research; instead, communication strategies to improve them are still underexplored. The studies by Carfora and Catellani [14] and Riverso and colleagues [15] address this topic with innovative approaches and results. Furthermore, recent research focused on consumer beliefs about alternative proteins, whereas few studies have been devoted to stakeholder beliefs. Amato and colleagues [16] propose a first systematic review of the existing studies on the topic. Noticeably, their results show that stakeholders’ beliefs are differently focused when compared to crucial beliefs attributed to consumers by previous research. The second review in the current Special Issue is provided by Andreani and colleagues [17] on the diverse plant-based alternatives to meat which are continuously introduced on the market, with a multi-disciplinary approach which considers technological and nutritional features as well as health-related factors and consumers’ acceptance. A plant-based food product is also under investigation in the experiment by Muhlhausler and colleagues [18]: results provide some optimistic considerations in regard to post-prandial satiety, which is another under-explored topic when it comes to possible alternatives to meat.

In sum, there are reasons to think that the market of meat substitutes may expand in the near future, depending on factors such as consumer acceptance, stakeholder beliefs, technical aspects, and communication. It is a topic on which paramount research has been conducted already; nevertheless, there are under-studied topics and challenges—that the current Special Issue aims to highlight—which still require a relevant effort by scholars.

In regard to future developments, research so far has been focused on antecedents which are often very specific and narrow. Connecting acceptance of meat substitutes with more general constructs such as identities, values, and lifestyles [14,19,20] may be a promising path for future research. Finally, it may be worth noting that almost all research conducted on alternative proteins so far employed explicit self-report measures for studying individuals’ attitudes and willingness to eat these foods (with few exceptions [21,22]). Nevertheless, implicit measures have been shown to also be predictive of behaviors in the food domain [23]. As such, future research may benefit studies employing multiple—explicit and implicit—measures to further understand consumers’ and stakeholders’ attitudes and behaviors.

## Figures and Tables

**Figure 1:**
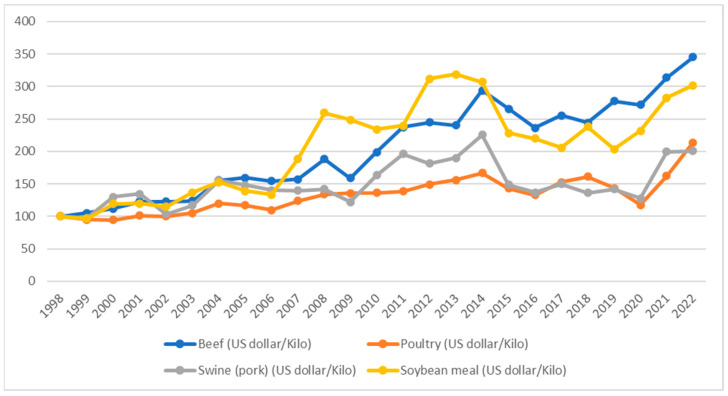
Main protein sources: price trends. Note. The y-axis reports price variation (rebased to 100). Source: authors’ own elaboration based on data by Indexmundi (www.indexmundi.com, accessed on 23 April 2023).

**Figure 2:**
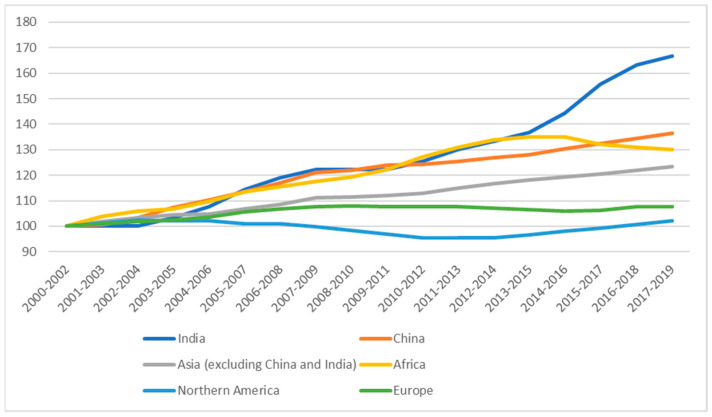
Trends in protein consumption. Note. The y-axis reports per capita protein consumption (rebased to 100). Source: authors’ own elaboration based on data by FAOSTAT (http://www.fao.org/faostat/en/ accessed on 23 April 2023).
